# HBsAg Loss as a Treatment Endpoint for Chronic HBV Infection: HBV Cure

**DOI:** 10.3390/v14040657

**Published:** 2022-03-22

**Authors:** Maryam Moini, Scott Fung

**Affiliations:** 1 Department of Medicine, University of Ottawa, Ottawa, ON K1Y 4E9, Canada; mmoini@toh.ca; 2 Department of Medicine, University of Toronto, Toronto, ON M5G 2C4, Canada

**Keywords:** hepatitis B virus (HBV), HBV cure, hepatitis B surface antigen (HBsAg), antiviral therapy

## Abstract

Despite the availability of effective vaccines and antiviral therapy over the past two to three decades, chronic hepatitis B virus (HBV) infection remains a major global health threat as a leading cause of cirrhosis and liver cancer. Functional HBV cure defined as hepatitis B surface antigen (HBsAg) loss and undetectable serum HBV DNA is associated with improved clinical outcomes in patients with chronic HBV infection. However, spontaneous loss of HBsAg is rare and occurs in only 1% of all HBsAg-positive individuals annually. Furthermore, the rate of functional cure with currently available antiviral therapy is even lower, <1% patients on treatment per year. Nonetheless, HBsAg loss has become the new target or therapeutic endpoint for antiviral treatment. Recently, there has been much excitement surrounding the development of novel antiviral agents such as small interfering RNA (siRNA), core assembly modulators (CAMs), nucleic acid polymers (NAPs) among others, which may be used in combination with nucleos(t)ide analogs and possibly immunomodulatory therapies to achieve functional cure in a significant proportion of patients with chronic hepatitis B. Novel assays with improved sensitivity for detection of very low levels of HBsAg and to determine the source of HBsAg production will also be required to measure efficacy of newer antiviral treatments for HBV cure. In this narrative review, we will define HBV cure, discuss various sources of HBsAg production, evaluate rates of HBsAg loss with current and future antiviral agents, review clinical factors associated with spontaneous HBsAg loss, and explore clinical implications of functional cure.

## 1. Introduction

Chronic hepatitis B virus (HBV) infection is a major health problem with an estimated global prevalence of about 3.9% or approximately 280 million individuals [1]. Patients with chronic HBV infection are at increased risk of progressive liver disease and hepatocellular carcinoma (HCC). Chronic HBV infection (CHB) remains a leading cause of liver cancer related mortality worldwide [2]. Introduction of HBV vaccination has led to a significant decrease in the incidence of acute and chronic infection and HCC [3,4,5]. Spontaneous hepatitis B surface antigen (HBsAg) seroconversion has been reported with an annual rate of approximately 1% and many studies have shown that HBsAg loss is associated with a decreased risk of cirrhosis and even HCC with excellent prognosis in patients younger than 50 years and no other liver related risk factors [2,6,7]. Long-term antiviral treatment which results in long-term suppression of viral replication has significantly reduced the risk of progression of liver disease; HCC risk can also be reduced with antiviral treatment but not entirely eliminated [8]. Therefore, the ultimate endpoint of antiviral therapy for HBV is HBsAg seroconversion, although this is rarely achieved with currently available nucleos(t)ide analogs (NA) and for most patients, long-term treatment (>10 years) may be needed. Thus, newer agents in combination with NA are urgently needed to reach this goal in a significant number of CHB patients. Table 1 summarizes the efficacy in terms of HBV DNA and HBsAg reduction of current and new agents for the treatment of chronic hepatitis B.

## 2. Quantitative HBsAg Assays

Quantitative serum HBsAg levels have been shown to correlate with intrahepatic cccDNA and serve as a surrogate marker for cccDNA transcriptional activity [9]. Currently available HBsAg assays are enzyme immunoassays (EIA) with a lower limit of quantitation of 0.05 IU/mL. However, highly sensitive chemiluminescent enzyme immunoassays (CLIA) with a lower limit of quantitation of 0.005 IU/mL are now available. In a study of 114 CHB patients who lost HBsAg by conventional EIA, 50% patients were found to have detectable HBsAg by high sensitivity CLIA. Patients receiving NA, those with cirrhosis and who were HBsAb-negative were more likely to have discrepant results, suggesting this subgroup requires ongoing monitoring even after HBsAg loss [10].

**Figure 1:**
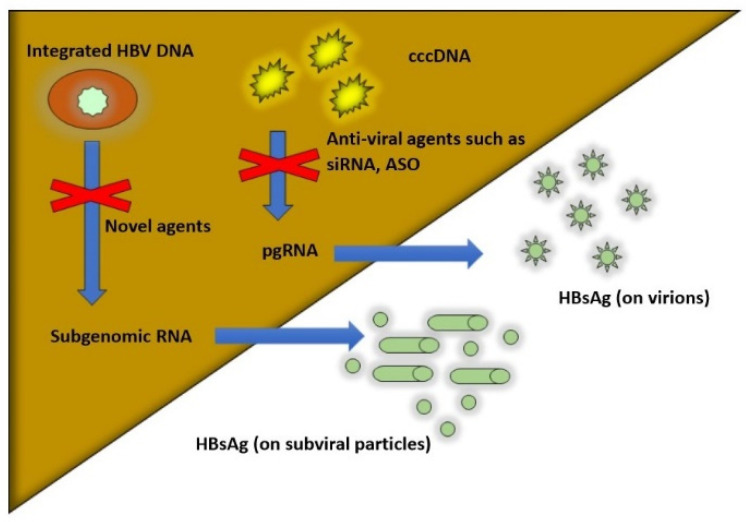
Different Sources of Hepatitis B s antigen (HBsAg) in the body. Red crosses show various pathways that are inhibited by different antiviral medications.

Current HBsAg assays are not able to differentiate between the different isoforms of HBsAg and their origin: cccDNA vs. integrated HBV DNA (see Figure 1). Low levels of HBsAg may be produced from integrated HBV DNA, particularly in HBeAg-negative patients. Furthermore, undetected quantities of HBsAg may be hidden or difficult to detect in HBsAg-HBsAb complexes. Vaccine escape mutants or a-determinant mutants (HBsAg-negative mutants) are quite rare and can be detected by most but not all enzyme-immunoassays for HBsAg [11].

## 3. Multiple Sources of HBsAg Production: cccDNA vs. Integrated DNA

As mentioned above, HBsAg is produced from different sources in the hepatocyte. HBsAg can be produced from covalently closed circular DNA (cccDNA) or from HBV DNA integrated into the hepatocyte nucleus. Integrated HBV DNA is believed to be the major source of HBsAg production among HBeAg-negative patients. Gene deletions and rearrangements are commonly seen in integrated HBV DNA sequences and result in production of truncated or fragmented forms of HBsAg, whereas cccDNA produces the full-length HBsAg [11,12]. In future, HBsAg assays will be able to distinguish between various isoforms of HBsAg to determine the degree of transcriptional activity of cccDNA and source of HBsAg production. Currently available NA have minimal impact on cccDNA levels and integrated HBV DNA levels. Newer antiviral agents in clinical development such as small-interfering RNA (siRNA) can target cccDNA transcriptional activity, as measured by reductions in quantitative HBsAg, HBV RNA, and HB core-related antigen (HBcrAg) levels. Novel agents that have not yet entered clinical testing include zinc finger nucleases that can cleave or epigenetically modify HBV DNA sequences leading to cccDNA silencing [13]. In addition, novel CRISPR-derived base-editing agents are under development, which can result in nonsense mutations in cccDNA and integrated HBV genomes [14]. Figure 1 depicts different sources of HBsAg production within the hepatocyte.

## 4. HBsAg Loss as a Treatment Endpoint

The definition of cure in HBV treatment is fundamental to determine the endpoint of currently available NA or interferon (IFN) treatment and to evaluate the success of future therapies in clinical development

i. Current treatment endpoints

Biochemical Response: Normalization of aminotransferases is a classic endpoint in HBV treatment. ALT normalization occurs in up to 70% patients after 1 year of NA therapy and increases with longer duration of therapy. Normalization of ALT has been shown to have a decreased risk of complications in long-term follow up [15]. However, aminotransferases are nonspecific for CHB and also lack sensitivity as a marker of disease severity [16]. In addition, the upper limit of normal for ALT varies between various laboratories and clinical trials [11].

Serologic Response: HBV serology has been used as another endpoint of treatment for CHB, particularly those who are HBeAg-positive at baseline. HBeAg-positive serology is associated with higher level of HBV replication and increased risk of HCC in older patients [17,18]. Spontaneous HBeAg loss and/or seroconversion has been associated with favourable prognosis and clinical outcome [19,20]. Although traditionally considered a surrogate endpoint, HBeAg seroconversion should be interpreted cautiously, as liver disease progression may still occur in patients who develop HBeAg-negative chronic hepatitis [16]. HBeAg seroconversion may be non-durable in those who received inadequate consolidation therapy [16].

Virologic Response: HBV DNA is the gold standard of care for monitoring response in those receiving NA therapy. Undetectable HBV DNA is the traditional endpoint and the first biomarker to show a response to treatment. In addition, biochemical and histologic improvement is seen with treatment-induced HBV DNA suppression [21]. A landmark study of untreated HBeAg-negative patients in Taiwan indicated that elevated HBV DNA was associated with a high risk of cirrhosis and hepatocellular carcinoma (HCC) [22]. Therefore, sustained viral suppression (undetectable or very low HBV DNA) is expected to reduce the risk of adverse liver outcomes. Indeed, a recent meta-analysis confirmed that NA therapy was associated with a more favourable prognosis [23]. Some studies have recommended persistently undetectable HBV DNA as an endpoint of NA therapy in HBeAg-negative patients, but this criterion for stopping treatment led to clinical relapse in over 50% patients requiring retreatment with NA [24]. Therefore, HBV DNA undetectability alone may not predict an off-treatment response compared to other biomarkers such as quantitative HBsAg levels.

Histologic Response: Histologic improvement can be seen in those who respond to long-term antiviral treatment. In a landmark study of paired biopsy samples from tenofovir disoproxil fumarate TDF-treated patients, regression of cirrhosis was seen in 75% patients after 5 years of continuous therapy [25]. In clinical practice, liver biopsies have been replaced by non-invasive test including serum fibrosis biomarkers and/or transient elastography (ultrasound/magnetic resonance) in the majority of patients. However, non-invasive tests (NIT) are mainly used to assess the severity of hepatic fibrosis without providing information on necroinflammatory activity and changes in NIT in CHB patients on treatment have not been well-studied.

ii. HBV Cure Definitions

Treatment-induced HBsAg loss is considered functional cure and resolution of chronic HBV infection. Several studies have documented favourable clinical outcomes following HBsAg loss or serocoversion at an early age (<50 years) in the absence of cirrhosis in those who had chronic HBV infection [26,27]. HBsAg loss rarely occurs in Asian patients receiving NA (<1% per year) but once achieved, it is usually sustained [28]. Seroconversion to hepatitis B surface antibody (HBsAb) occurs in a minority of patients who lose HBsAg on treatment. However, for patients receiving NA, HBsAg loss without gain of HBsAb is an acceptable endpoint of treatment and associated with good outcomes [26,29].

In contradistinction, occult HBV infection defined as HBsAg-negative in the presence of low-level HBV DNA (<2 log IU/mL) [30]. Unlike functional cure, occult HBV infection has been associated with progressive liver disease and hepatocellular carcinoma.

### 4.1. Functional Cure

At a recent meeting of the European Association for the Study of the Liver (EASL) and the American Association for the Study of Liver Diseases (AASLD) in 2019 [31], functional cure was defined as HBsAg loss or <0.05 IU/mL (with or without seroconversion to HBsAb) and undetectable HBV DNA (or <10 IU/mL) maintained for a minimum of 6 months after treatment discontinuation. Currently, sustained HBsAg loss confirmed on two occasions at least 6 months apart in conjunction with undetectable HBV DNA is considered the best surrogate of functional cure [31]. Functional cure with NA therapy occurs at a low rate (<10% patients after 10 years of continuous TDF therapy), but is still considered a feasible endpoint of treatment.

### 4.2. Partial Cure

With partial cure, HBsAg remains positive, HBeAg-negative with undetectable or low levels of serum HBV DNA and inactive liver histology [31]. Partial cure is a more realistic endpoint of current NA in most patients receiving NA and has been reported in up to 83% HBeAg-negative patients who have been treated with NA therapy for up to 10 years [32].

### 4.3. Sterilizing Cure

Sterilizing cure is beyond what can be achieved with currently available treatment. The clinical scenario would correlate with a patient who had never been infected with HBV: HBsAg-negative, HBsAb-positive or negative, HBeAg-negative, undetectable serum HBV DNA, undetectable covalently closed circular DNA (cccDNA), and no integrated HBV DNA and no histologic evidence of progressive liver disease [31]. Even immune competent adult patients who have recovered from acute HBV infection cannot attain a state of sterilizing cure. In order to achieve this stringent endpoint, future agents that can remove cccDNA and excise integrated HBV DNA will be required.

i. When to Stop NA using HBsAg levels

Expert treatment guidelines have recommended treatment discontinuation in noncirrhotic patients who have lost HBsAg [33,34]. However, this may take decades of continuous treatment to achieve and for many, NA therapy will be lifelong. Is it therefore possible to consider treatment withdrawal before HBsAg seroclearance is achieved? Quantitative HBsAg levels may be able to guide the decision regarding when to stop treatment. In a systematic review of over 1700 patients treated with NA, HBsAg <100 IU/mL at the end of treatment was associated with lower rates of relapse off-treatment [35]. Similarly, in a large international retrospective cohort study of 1550 patients treated with NA, Caucasian patients and those with HBsAg <100 IU/mL at the end of treatment had a higher probability of HBsAg loss >30%. Nonetheless, hepatic decompensation and HCC were reported in a small number of patients and ongoing monitoring post treatment is required. Based on the above findings, noncirrhotic HBeAg-negative patients who achieve HBsAg < 100 IU/mL (Asians) or <1000 IU/mL (Caucasians) were suitable candidates for treatment discontinuation before HBsAg loss [36].

**Figure 2:**
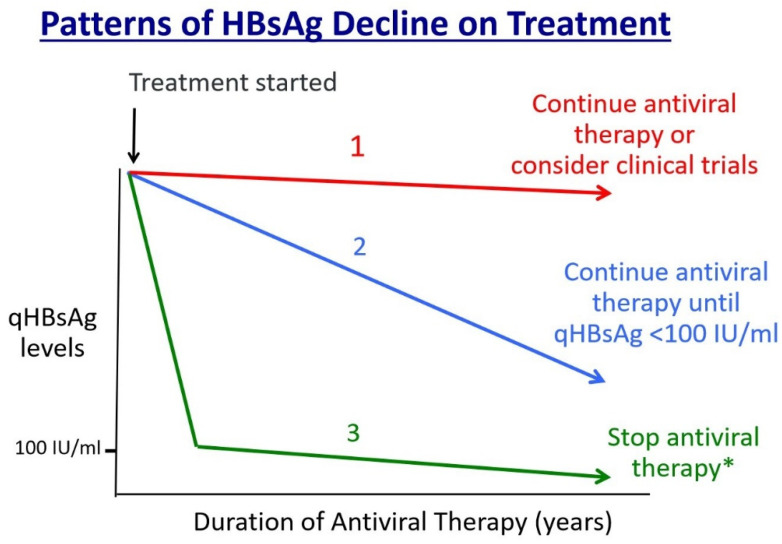
Different patterns of decline in quantitative HBsAg levels in patients with chronic hepatitis B on antiviral treatment and management plan for each pattern. * In HBe antigen negative, non-cirrhotic patients.

The different patterns of decline in quantitative HBsAg levels are depicted in Figure 2. Patients with little or no drop in qHBsAg levels should continue antiviral therapy indefinitely or consider participation in clinical trials of HBV cure, if appropriate (pattern 1). Those who demonstrate a consistent decline in qHBsAg should be encouraged to continue treatment, even if for many years, until qHBsAg levels drop below 100 IU/mL to maximize the chances of an off-treatment response (pattern 2). Lastly, for those who show a rapid decline in qHBsAg to below 100 IU/mL, antiviral treatment can be stopped after 12 months of consolidation therapy in those who have also lost HBeAg and have no evidence of cirrhosis (pattern 3) [36].

## 5. Rates of HBsAg Loss

i. Spontaneous clearance of HBsAg

Spontaneous HBsAg loss occurs at rate of approximately 1% per year and typically occurs in HBeAg-negative patients with low viremia and inactive disease (inactive carrier state) [37,38]. Previous reports suggested higher rates of spontaneous HBsAg loss in endemic areas comparing with non-endemic areas [39,40]. However, recent studies and a systematic review were not supportive of this finding [38,41]. In a separate systematic review, the rate of spontaneous HBsAg clearance in cirrhotic patients was shown to be comparable to that of non-cirrhotic patients (1.1%) [38]. However, loss of HBsAg at a younger age is expected to lead to a better liver outcome.

ii. Treatment- induced HBsAg Loss

HBsAg loss is rarely achieved with the currently available treatment among Asian patients. The highest rate of HBsAg loss is seen among HBeAg-positive patients receiving PEG-IFN for 1 year and lower rates are seen in HBeAg-negative patients treated with NA for many years [33]. Predictors of IFN response include HBeAg positive status, HBsAg decline on treatment, HBV genotype A or B, lower HBV DNA levels and absence of cirrhosis pre-treatment [42]. Approximately 5–10% patients will lose HBsAg during treatment or during follow-up post-PEG-IFN. Higher rates of HBsAg loss were reported in a large prospective study of HBeAg-positive patients who received a combination of PEG-IFN plus TDF compared to either monotherapy [43]. Once HBsAg loss is achieved, the durability is generally thought to be high [44,45], irrespective of HBsAb status [44]. A large international study reported a cumulative rate of HBsAg was reported to be 13% in HBeAg-negative patients 4 years after treatment discontinuation. Predictors of HBsAg loss were Caucasian race and younger age of HBsAg loss. On the other hand, Asians and older patients were more likely to require retreatment with NA during follow-up [36].

Oral NA therapy is preferred by the vast majority of patients requiring treatment as it is highly effective, and very safe long-term. First line agents in North America include entecavir (ETV) and the two prodrugs of tenofovir, tenofovir disoproxil fumarate (TDF) and tenofovir alafenamide (TAF), all of which are highly potent and have a high barrier to genotypic resistance. During long-term treatment with ETV, TDF or TAF, the vast majority of HBeAg-positive and HBeAg-negative patients become HBV DNA undetectable. HBeAg seroconversion during NA therapy occurs in 40–50% HBeAg-positive patients after 4–5 years of continuous therapy. However, HBsAg and cccDNA are minimally impacted by NAs, which contribute to viral persistence and relapse following treatment withdrawal. It is estimated that the annual rate of HBsAg loss is well below 1% [28]. In fact, the median rate of HBsAg reduction over 5 years of ETV was approximately 0.13 log10 IU/mL per year [36]. This means that most patients will require long-term (>10 years) NA therapy to achieve significant declines in HBsAg and perhaps HBsAg loss.

iii. Novel therapies for HBV cure

There are several different therapeutic approaches to achieve functional HBV cure. The virologic approach aims at elimination of cccDNA through blockade of viral replication at multiple steps. On the other hand, the immunologic approach is based on host immune modulation leading to HBsAg loss with agents such as PEG-interferon, toll-like receptor agonists, PD1/PD-L1. A detailed discussion of these agents is beyond the scope of this review. It is likely that future successful therapies will require a combination of the two approaches to achieve HBV cure: antiviral agents will reduce viral antigen production, while the immune modulators will enhance or restore host immunity against HBV [46]. New clinical trials are currently testing various combinations of antiviral agents and immune modulators for a finite duration and will measure HBsAg loss as the primary efficacy endpoint to determine rates of HBV cure.

Novel direct-acting antivirals (DAAs) against hepatitis B are classified based on their action targets into two groups: (1) HBV replicative inhibitors and (2) HBV translation inhibitors [47]. Replicative inhibitors include entry inhibitors, capsid assembly modulators, nucleic acid polymers (NAPS) and currently available NAs. In general, these agents have shown only modest efficacy in reducing quantitative HBsAg levels (<1 log IU/mL), partly due to short duration of treatment when combined with long-term NA therapy in treatment-naïve or virally suppressed patients. These agents have been safe and well-tolerated in phase one and two trials. Of note, NAPs can inhibit both virus particle production and their secretion [48]. This class of replicative inhibitor has shown the most potent HBsAg reduction when combined with PEG-IFN and/or TDF with HBsAg loss reported in up to 40% patients with chronic hepatitis B infection in small clinical trials.

Novel translation inhibitors target translation of various HBV gene products, thereby reducing production of viral proteins or antigens. This class of antivirals includes small-interfering RNAs (siRNAs) and antisense oligonucleotides (ASOs) and locked nucleic acid oligonucleotide (LNA) [47,49]. siRNA and ASO have shown moderate HBsAg reduction in short-term clinical trials, approximately 1–2 log IU/mL after 24 weeks of treatment with or without NA combination. Although generally safe and well tolerated, significant ALT flares have been reported during or after treatment withdrawal using siRNA and ASO, which requires close monitoring and further study in larger trials. The relative antiviral potency of various novel antiviral agents from early clinical studies is shown in Table 1.

## 6. Predictors of HBsAg Loss

i. Baseline HBsAg levels

It is not surprising that a higher rate of spontaneous HBsAg loss is seen in patients with lower quantitative HBsAg level [50,51]. HBsAg titer compared to HBV DNA level is a more accurate predictor of spontaneous HBsAg loss in chronic hepatitis B patients [52]. Declining levels of HBsAg reflect enhanced innate immunity against HBV replication and eventual HBsAg loss.

Baseline HBsAg level was shown to predict the response to PEG-IFN alpha 2a in patients with HBeAg-negative patients [24]. Lower baseline HBsAg titre was predictive of HBsAg loss after 48 weeks of PEG-IFN in a study of inactive carriers in China. Furthermore, lower baseline HBsAg titer was also associated with prolonged sustained response to treatment [53,54,55]. In those receiving NA, lower baseline HBsAg levels in HBeAg-positive patients was associated with HBsAg loss [56,57].

ii. HBsAg decline

Studies suggest that monitoring of quantitative HBsAg during treatment can be used as a predictor of response to treatment [58]. A decline of >2 log IU/mL in HBsAg level at months 6 of treatment with TDF was found to be a predictor of HBsAg loss in HBeAg-positive patients co-infected with HIV [59]. Furthermore, rapid early decline in HBsAg > 1 log IU/mL at week 24 of treatment with TDF in HBeAg-positive patients was predictive of HBsAg loss in Caucasian patients infected with genotypes A or D [60]. In a three-year follow up study of CHB patients treated with TDF, those who achieved HBsAg loss showed a greater decline in HBsAg titer at week 24 of therapy [61]. Unfortunately, quantitative HBsAg levels are not a reliable predictor of response in HBeAg-negative patients.

Similarly, HBsAg loss on PEG-IFN was more likely to occur in those who had HBsAg decline >1 log IU/mL at week 12 [46] or HBsAg < 2 log IU/mL at week 12 [55,62]. Those who achieved HBsAg reduction by >2 log IU/mL at week 24 on therapy were more likely to go to HBsAg loss. In contrast, those who do not achieve this response, should discontinue treatment and avoid further side effects, since the chance of HBsAg loss was minimal. Similarly, low HBsAg level at the end of treatment was associated with a higher sustained virologic response to PEG-IFN alpha 2a [63].

iii. HBV Genotype

Hepatitis B genotype may also influence the response to treatment. In a large randomized study of PEG-IFN, higher rates of HBsAg loss were observed in patients with genotype A compared to genotype D and E [64]. Similarly, PEG-IFN treatment showed a higher rate of response among genotype B compared to genotype C patients [65,66]. However, the impact of HBV genotype on response to NA therapy is less well-established. There are conflicting results with higher response rates to lamivudine among genotype B compared to genotype C patients [67], but the impact of genotype on response to lamivudine was not confirmed in the other studies [68,69]. For other NAs, there appears to be no significant difference in treatment response among different HBV genotypes [69,70].

iv. HBV DNA

Lower baseline HBV DNA level is associated with higher rate of HBsAg loss in patients receiving treatment with PEG-IFN [71,72]. Also, HBV DNA decline during treatment with PEG-IFN may also predict response to treatment [73]. However, it is unclear if HBV DNA is predictive of HBsAg loss among those treated with NA. In one particular study from Taiwan, lower HBV DNA level was a predictor of HBsAg loss or seroconversion on NA [74]. Although baseline HBV DNA is a predictor of virologic response to NA [75], it was unable to predict clinical relapse, the need for retreatment or HBsAg loss after NA treatment withdrawal in recent stop-treatment studies [24,76].

v. HBeAg status

Among 266 HBeAg-positive patients treated with TDF for 5 years, HBsAg loss was observed in 23 (9%) patients [60]. In that particular study, predictors of HBsAg loss included Caucasian race, genotype A or D and shorter duration of HBV infection of <4 years and HBeAg-positive status. HBsAg decline >1 log IU/mL at week 24 was also predictive of HBsAg loss. In that study, rapid HBV DNA decline occurred initially, followed by HBsAg decline over 1 log IU/mL by week 24, then HBeAg loss before eventual HBsAg loss.

vi. ALT levels

Higher pre-treatment ALT level and lower HBV DNA have been associated with higher HBsAg seroconversion rate among HBeAg-positive patients treated with PEG-IFN [71]. In HBeAg-positive patients treated with lamivudine, HBsAg seroconversion was shown to be higher among those with baseline higher ALT level [77]. Indeed, in early phase clinical trials of agents in development for HBV cure such as siRNA and NAPs in combination with PEG-IFN, ALT flares have been reported following HBsAg decline in a limited number of patients who achieved functional cure [78]. Taken together, these studies suggest restoration of cell-mediated immunity against HBV is reflected by vigorous necroinflammatory activity in the liver, manifested by serum ALT elevation which can lead to HBsAg loss.

vii. NAFLD/Hepatosteatosis

In patients with chronic hepatitis B, non-alcoholic fatty liver (NAFL) is often detected on abdominal ultrasound for the purpose of HCC screening. The prevalence of NAFL in CHB patients is up to 33% and is related to the increasing prevalence diabetes mellitus, obesity, hypertension and chronic kidney disease amongst aging CHB patients [79,80]. Although hepatosteatosis is very common, progression to non-alcoholic steatohepatitis (NASH) occurs in 20–25% patients. NASH presents as a mild to moderate elevation in ALT in patients with low levels or undetectable HBV DNA and quantitative HBsAg is often low. In a large liver biopsy study of almost 350 patients treated with TDF, a lack of regression in fibrosis on biopsy at 5 years was associated with the presence of high BMI, diabetes, and presumably NASH at baseline [25].

Although the presence of NAFL in CHB patients does not adversely affect the response to NA or IFN [81], metabolic risk factors have been associated with increased fibrosis and cirrhosis, [82,83]. To date, several studies have examined the interaction between NASH and HBV with conflicting results. In a retrospective study of over 1000 CHB patients referred to tertiary liver clinics, those with NASH had increased risk for development of advanced hepatic fibrosis and HCC compared to those with CHB alone [84]. However, no association was found between hepatosteatosis and liver complications in CHB patients without advanced fibrosis. On the other hand, in another large retrospective study, CHB patients with NAFLD had a lower risk for development of cirrhosis and were more likely to lose HBsAg irrespective of antiviral treatment [85]. However, the mechanism for this potentially protective effect of NAFLD in CHB remains unexplained and requires further study.

Table 2 summarizes the factors affecting HBsAg loss with treatment in chronic hepatitis B.

## 7. Clinical Benefits of HBsAg Loss

Patients who have spontaneously lost HBsAg and HBV DNA are those who have achieved strong immune control over the infection and generally have a very good prognosis if HBsAg loss occurs before the age of 50–60 years and in the absence of cirrhosis. However, HBsAg-positive patients with inactive chronic hepatitis B infection (inactive carriers) are also thought to have an overall good prognosis compared to those with active hepatitis B infection [33], which may make the comparison between this group and those who have lost HBsAg difficult. However, HBsAg loss is strongly associated with a reduced risk of long-term adverse clinical outcomes observed among CHB patients regardless of the presence of cirrhosis [86]. However, the magnitude of the clinical benefit is greater in those who are noncirrhotic and younger than 50–60 years old at the time of HBsAg loss—whether it is spontaneous or treatment-induced. In a recent large retrospective study, there was no significant difference in the risk of HCC and clinical outcomes between spontaneous and NA-induced HBsAg seroclearance [87].

i. HCC reduction

A recent systematic review and meta-analysis showed a reduced risk of HCC among all age groups with spontaneous or treatment induced HBsAg seroclearance [86]. Furthermore, spontaneous seroclearance of HBsAg in noncirrhotic patients was associated with decreased risk of HCC [88]. Similarly, treatment-induced HBsAg seroclearance was also associated with a lower risk of HCC development both in cirrhotic and non-cirrhotic patients [27]. The risk was even more pronounced in those without cirrhosis at baseline [89]. However, other studies have indicated that the risk of HCC remains unchanged after HBsAg loss in patients over the age of 50 years and in those with pre-existing cirrhosis. Therefore, ongoing HCC screening after HBsAg loss is advised in this subset of patients [90].

ii. Reduced risk of cirrhosis and need for liver transplant

Spontaneous HBsAg loss was reported to be associated with decreased incidence of cirrhosis, particularly when it occurred early in life [38]. However, in a study of CHB patients treated with 5 years of TDF, HBsAg loss was not associated with regression of cirrhosis, suggesting that longer follow-up is needed to demonstrate this benefit [25]. A reduced risk of liver transplantation or death was observed in those with spontaneous or treatment-induced HBsAg loss in several studies [86,91]. This is likely due to reduction in complications of decompensated liver disease such as ascites, clinically significant portal hypertension, and development of HCC.

iii. Durability of HBsAg loss

HBsAg loss after treatment with NA or PEG-IFN was demonstrated to be durable independent of consolidation treatment duration and HBsAb seroconversion [36]. The durability of NA-induced HBsAg seroclearance is comparable with that of spontaneous seroclearance [73]. In a retrospective study of almost 2000 patients in Korea, 98% patients with spontaneous or NA-induced HBsAg loss showed a durable response over >5 years of follow-up. A small minority underwent HBsAg seroreversion, but most of these patients re-cleared HBsAg during follow up without re-treatment [87].

## 8. Long-Term Monitoring after HBsAg Loss

Spontaneous or treatment-induced HBsAg loss has been shown to be durable and liver-related complications are reduced. The risk of HBsAg seroreversion is very low following documented HBsAg loss. Therefore, routine monitoring of HBsAg with liver enzymes can be performed annually only, since HBsAg reappearance is likely to be very low. Even though HBsAg loss is associated with a lower risk of HCC, it is not entirely eliminated. Risk factors for HCC after HBsAg loss include older age and the presence of cirrhosis pre-dating HBsAg loss. Therefore, ongoing screening for HCC is recommended for cirrhotic patients over the age of 50–60 years at the time of HBsAg loss. However, the optimal frequency (e.g., every 6–12 months or even less frequently) has not been established [7,87].

## 9. Summary

HBV cure forms a key component of the global efforts for viral hepatitis elimination by the year 2030. Functional cure of chronic hepatitis entails loss of HBsAg with undetectable HBV DNA that is sustained for at least 6 months following treatment discontinuation. HBsAg loss is a relatively rare event in the natural history of chronic HBV infection, occurring in only 1% patients per year. Unfortunately, HBsAg loss on NA occurs at an even lower rate <0.5% per year, suggesting that HBV cure is even more difficult to achieve in those who have active hepatitis requiring antiviral treatment. However, among PEG-IFN treated patients who were treated for 1 year, 10% patients achieved functional cure 3 years after treatment discontinuation. Therefore, various strategies to maximize the rate of HBsAg loss, include continuous long-term therapy >10 years resulting in HBV cure in up to 10% Asian patients. In contrast, treatment withdrawal has met with some success in approx. 20% Caucasian patients, although the same result was not seen among Asian patients who discontinued long-term NA. Predictors of HBsAg loss with PEG-IFN include HBV genotype A or B, rapid HBsAg reduction on treatment, HBeAg-positive status and lower HBV DNA levels. Unfortunately, there are few reliable predictors of HBsAg loss on NA but may include HBV genotype A, HBeAg-positive status and Caucasian race. Interestingly, hepatosteatosis on abdominal ultrasound has been identified in some studies as a predictor for expedited HBsAg loss. There are several clinical benefits that occur following HBsAg loss including remission of liver disease and a mitigated risk of HCC development in younger non-cirrhotic patients.

In order to achieve functional cure among a meaningful proportion of CHB patients, development of novel classes of antiviral agents is currently underway. These include NAPS, CAMs, siRNA, and ASO in addition to immune modulatory agents such as toll-like receptors (TLR) agonists, therapeutic vaccines and PD1-PD-L1 agents, which are beyond the scope of this review. These agents have shown great promise in achieving HBsAg loss in early clinical trials. However, many questions remain to be answered including optimal combinations of agents, optimal duration of therapy, durability of response and most importantly, safety and tolerability. Nonetheless, there remains much excitement and optimism in the field of HBV treatment and major advances are expected that will lead to persistent HBsAg loss or functional cure in many CHB patients.

## Figures and Tables

**Table 1:** Effect of nucleos(t)ide analogs, PEG-IFN, and direct-acting antivirals in clinical development for chronic hepatitis B treatment.

Agent & Mode of Action	Drug(s)	Delivery	Phase of Development	Change In:	
				HBV DNA	HBsAg
Nucleos(t)ideAnalogs (NA)	TDF, TAF ETV	Oral	Approved	+++	+
Interferons	PEG-IFNα	Subcutaneous injection	Approved	++	++
Capsid assembly modulator (CAM)	Vebicorvir (H0731) ^†^, JNJ-6379 ^†^, EDP-514, RG7907, ABI-H3733, ALG-000184, AB-836	Oral	I & II	+++	+
Small interfering RNA (siRNA)	JNJ-3989 (ARO-HBV), VIR-2218 ^, AB-729, RG6346	Subcutaneous injection	II	++	++
Antisense oligonucleotide (ASO)	GSK 3228836, GSK 3389404	Subcutaneous injection	II	++	+++
Nucleic acid polymer (NAP)	REP-2139 ^††^ REP-2165 ^††^ ALG-10133	Intravenous infusion or subcutaneous injection	II	+++	+++

ETV, entecavir; HBsAg, hepatitis B surface antigen; HBV, hepatitis B virus; PEG-IFN, pegylated interferon; TAF, tenofovir alafenamide; TDF, tenofovir disoproxil fumarate. + Minimal: <1 log10 IU/mL decline at nadir within approximately 6 months; ++ Moderate: 1–2 log10 IU/mL decline at nadir within approximately 6 months; +++ Significant: >2 log10 IU/mL decline at nadir within approximately 6 months; ^†^ Significant suppression of HBV DNA seen when CAMs are combined with NA; ^††^ Significant suppression of HBV DNA and HBsAg when NAPs are combined with PEG-IFN + NA; ^ Significant suppression of HBV DNA and HBsAg when VIR2218 is combined with PEG-IFN.

**Table 2:** Factors associated with HBsAg loss on treatment.

Lower Quantitative HBsAg Level
Rapid decline in HBsAg level within 6 months of initiation of treatment
Lower baseline HBV DNA level
Higher baseline ALT level
HBV genotype ^†^

ALT, alanine transaminase; HBs Ag, hepatitis B surface antigen; HBV, hepatitis B virus; ^†^ Conflicting results based on the type of treatment.

## Data Availability

Not applicable.
